# ReMo-SNPs: a new software tool for identification of polymorphisms in regions and motifs genome-wide

**DOI:** 10.1017/S0016672315000051

**Published:** 2015-04-17

**Authors:** LISETTE GRAAE, SILVIA PADDOCK, ANDREA CARMINE BELIN

**Affiliations:** 1 Department of Neuroscience, Karolinska Institutet, Retzius väg 8, 171 77 Stockholm; 2 Rose Li and Associates, Inc., Bethesda, MD, USA

## Abstract

Studies of complex genetic diseases have revealed many risk factors of small effect, but the combined amount of heritability explained is still low. Genome-wide association studies are often underpowered to identify true effects because of the very large number of parallel tests. There is, therefore, a great need to generate data sets that are enriched for those markers that have an increased *a priori* chance of being functional, such as markers in genomic regions involved in gene regulation. ReMo-SNPs is a computational program developed to aid researchers in the process of selecting functional SNPs for association analyses in user-specified regions and/or motifs genome-wide. The useful feature of automatic selection of genotyped markers in the user-provided material makes the output data ready to be used in a following association study. In this article we describe the program and its functions. We also validate the program by including an example study on three different transcription factors and results from an association study on two psychiatric phenotypes. The flexibility of the ReMo-SNPs program enables the user to study any region or sequence of interest, without limitation to transcription factor binding regions and motifs. The program is freely available at: http://www.neuro.ki.se/ReMo-SNPs/

## 1. Introduction

Recent advances in high-throughput sequencing and genotyping techniques have enabled researchers to generate unprecedented amounts of genomic data. These efforts have led to the identification of more than 60 million single nucleotide polymorphisms (SNPs) (Frazer *et al.*, 2007). Information about these markers has been gathered in the National Center for Biotechnology Information (NCBI) Database of Single Nucleotide Polymorphisms (dbSNP), which holds information about their location, alleles and frequencies (Sherry *et al.*, 2001). Since the coding sequences make up less than 2% of the human genome (Vernot *et al.*, 2012), the vast majority of the identified SNPs are placed in non-coding DNA sequences, for which the function is not always evident.

While some rare diseases, such as sickle cell anaemia, cystic fibrosis and haemophilia, are caused by a single mutation in a coding DNA sequence, most diseases have a more complex, genetic component, likely involving a considerable variety of genetic risk factors. Recent genome-wide association studies (GWAS) have identified several thousand SNPs associated with a large number of complex traits and phenotypes. A majority of these associated SNPs are located in non-transcribed regions of the genome, which makes it harder to explain the underlying disease mechanism (Schaub *et al.*, 2012; Bulik-Sullivan *et al.*, 2013). It is, however, well known that non-coding sequences comprise important regulatory sequences, such as transcription factor binding regions, which play an important role in gene regulation. Recent large-scale efforts such as the ENCODE and the GTEx projects have contributed greatly to our understanding of these regions and their role in regulating gene transcription levels (The Encode Project Consortium, 2012; Lonsdale *et al.*, 2013).

Transcription factors usually recognize and bind to specific DNA sequences called motifs. The motif may be located in close proximity to or even within the gene it regulates. It can, however, also be found at a considerable distance from the gene (Lin *et al.*, 2007). The binding of transcription factors acts as a molecular switch and regulates the timing and amount of gene transcription. Mutations in these regulatory sequences that are introduced by SNPs occurring within the motif may therefore have a major impact on gene function and could in many cases contribute to disease risk, onset and/or severity.

Several previous attempts have been made to construct tools to search for SNPs placed within regulatory DNA sequences. FASTSNP (Yuan *et al.*, 2006), PupaSuite (Conde *et al.*, 2006), SNPlogic (Pico *et al.*, 2009) and regSNPs (Teng *et al.*, 2012) are all examples of tools to identify and analyse SNPs in transcription factor binding sites. One common limitation of these tools is that they depend on already existing knowledge of binding models for transcription factors gathered in different databases. Several of these tools also include a scoring system for SNP prioritization based on previously reported knowledge of transcription factor binding regions and disease correlations. In addition, many of these tools can only analyse one region or one gene at a time.

In a previous study (Graae *et al.*, 2012), we studied estrogen receptor (ER) binding variation genome-wide. We were interested in combining the *in silico* results from the motif analysis with evidence from experimental studies that had mapped ER binding across the genome. We developed several Perl scripts to aid us with the computational tasks during that study. We found this method of selecting SNPs for an association study very fruitful and to further automate and simplify this process, we have now developed a computational tool to search for SNPs in any region and/or motif of interest genome-wide. We have included results on several such analyses in this work and also studied the resulting SNPs of interest in GWAS on two psychiatric phenotypes.

A unique feature of ReMo-SNPs is the possibility to search for SNPs in both regions and motifs of interest, which enables the user to combine *in silico* identified motif data with functional *in vitro* or *in vivo* experimental data. In addition, the program can provide a list of which of the SNPs of interest are included in the user-provided material of genotyped SNPs. The program further maximizes the number of available data points for the GWAS study by identifying genotyped SNPs in high linkage disequilibrium (LD) according to a user-defined threshold for the interesting SNPs that have not been genotyped directly. Thus, the output files with the interesting and genotyped region and/or motif SNPs generated by the ReMo-SNPs program are ready to be used in a following GWAS study.

The flexibility of ReMo-SNPs makes it easy to adapt to different projects and research questions. This tool will allow scientists to carry out studies in any region or motif of interest genome-wide, without limitation to transcription factor binding regions. By using DNase I hypersensitivity sites as regions in ReMo-SNPs, for example, one is able to study several classes of *cis*-regulatory elements including promoters, enhancers, insulators, silencers and locus control regions. Another important field of research where the ReMo-SNPs program could be of great use is in the study of epigenetic changes of the genome. SNP differences in regions with histone modification or DNA methylation may easily be studied by using the ReMo-SNPs program (Pellegrini & Ferrari, 2012). Other types of input regions for the ReMo-SNPs program might for example be the genomic regions for several genes involved in a specific pathway of interest.

We believe that researchers will find the unique features of ReMo-SNPs useful when integrating *in silico* and functional data and using the derived information to analyse real-world association data. The program is freely available online and can be downloaded at: http://www.neuro.ki.se/ReMo-SNPs/

## 2. Materials

### (i) Individuals

Two data sets have been used in this study; one included individuals diagnosed with major depression (MD) and healthy controls and the other individuals diagnosed with bipolar disorder (BP) and healthy controls. The numbers of individuals included in the final data sets for the association analyses are shown in Table 1.

**Table 1.** Number of individuals included in the final data sets for the association analyses.

	Total		Women		Men	
	Cases	Control	Cases	Control	Cases	Control
MD	1727	1758	1200	1076	527	682
BP	964	998	487	490	477	508

The Netherlands Study of Depression and Anxiety (NESDA; http://www.nesda.nl), a longitudinal cohort study, has collected the MD material. Cases were recruited from mental health care organizations, primary care and community samples. Inclusion criteria were a lifetime diagnosis of Diagnostic and Statistical Manual of Mental Disorders, 4th Edition, major depression disorder as diagnosed by the Composite International Diagnostic Interview psychiatric interview, age 18–65 years and self-reported Western European ancestry. The control subjects, matched for age and sex and also of Western European ancestry, were derived from the Netherlands Twin Register (NTR; http://www.tweelingenregister.org), which has collected longitudinal data from twins and their families since 1991. After the first quality control analyses samples were excluded from the study if they failed quality criteria such as: uncertain linkage between genotype and phenotype, genomic outliers, such as too high genome-wide homozygosity (~75%), samples with contamination, failed genotyping or excessive missing genotype data (<25%) (Boomsma *et al.*, 2008).

The National Institute of Mental Health Human Genetics Initiative (NIMH GI; http://nimhgenetics.org/) has collected and characterized samples from individuals, of European ancestry, diagnosed with BP for the Bipolar Disorder Consortium (Bipolar consortium). The cases were interviewed with the Diagnostic Interview for Genetic Studies (DIGS) and diagnosed with a standard best estimate final diagnosis (BEFD) procedure. The control subjects, also of European ancestry, were collected separately through a NIMH-supported contract mechanism between Dr Pablo Gejman and Knowledge Networks, Inc. Average age at onset for the cases was 19 years and average age at study start for the controls was 52 years. Individuals that did not meet quality control criteria, such as low call rate, excessively high or low heterozygosity, incompatibility between reported gender and genetically determined gender or unexpected familial relationships, were removed from the study (Smith *et al.*, 2009).

### (ii) Genotype data and quality control

Genome-wide genotype data for individuals in the two data sets were obtained from the Genetic Association Information Network (GAIN). Written informed consent had been obtained by the original investigators from all participants in the study. The study was conducted in accordance with GAIN and the investigators.

The Perlegen GWAS platform was used for genotyping of the MD sample, which was conducted by Perlegen Sciences (Mountain View, CA, USA), and has been described elsewhere (Sullivan *et al.*, 2009). The Study Accession ID for the MD sample is phs000020.v2.p1.

The Broad Institute Center for Genotyping and Analysis (http://www.broad.mit.edu/node/306) used the Affymetrix Genome-Wide Human SNP Array 6·0 platform for genotyping the BP samples, which has been described by Smith *et al*. (2009). The dbGaP Study Accession ID for the bipolar study is phs000017.v3.p1.

We performed additional quality control steps and excluded individuals if the missing rate/person was >0·1 and SNPs with a Hardy-Weinberg equilibrium p-value of ⩽0·0001 in the controls, a minor allele frequency of <0·01 or if missing genotypes were >0·05 (Graae *et al.*, 2012).

## 3. Methods

The ReMo-SNPs Perl script is a computational tool to search for polymorphic markers (SNPs) in user-specified regions and/or motifs genome-wide. The program and all of the tools are fully available online at http://www.neuro.ki.se/ReMo-SNPs. A Perl interpreter is required to run the script. Most modern Unix/Linux/OS X machines come with a Perl interpreter, in which case no additional installation is required.

Here we describe the definitions of the different files needed to run the ReMo program:

### (i) Definitions

#### (a) Regions and motifs

Regions are genomic areas of interest, while motifs refer to the specific nucleotide patterns that transcription factors recognize. The user defines which genomic regions the program should search through. In our example we included experimentally validated binding regions for three different transcription factors: the glucocorticoid receptor (GR), the peroxisome proliferator-activated receptor (PPAR) and the vitamin D receptor (VDR). The user can also define a motif of interest that the program should search for, e.g. the specific binding motif for each transcription factor. The program searches for the motif of interest as well as the reverse complement sequence in a step-wise manner, going through each one of the downloaded nucleotide sequence files (FASTA files) moving one nucleotide at a time.

The BED file contains information about genomic regions of interest. The user may assign a score to highlight regions of special interest. The default score value is set to 1. The BED file should thus contain three to five columns with the following information: chromosome, start position (bp), end position (bp), name (optional) and score (optional).

#### (b) Region score

The region score (stated in the last column of the BED file) is used to prioritize regions of interest, whether the motif is present within the region or not. The user can specify a score for each genomic region. On the command line, the user can then specify thresholds for the region score. Each region with a score above the threshold will be included in the analysis, even when no transcription factor binding motif is found within the region. The region score option thus allows the user to ensure that regions with strong experimental support are included in the analysis, whether or not the *in silico* analysis suggests the presence of a binding site.

The motif file is a text file with the motif of interest written in International Union of Pure and Applied Chemistry (IUPAC) code. ReMo-SNPs can currently analyse one motif at a time. Therefore, only one motif per file is currently allowed.

FASTA files provide a simple format to store nucleotide sequences. They contain a header and, beneath that, the genetic code in a plain format, letter by letter. The ReMo-SNPs program uses one FASTA file for each chromosome.

The HapMap file provides physical positions (chromosome and bp) for SNPs identified in the HapMap project.

The ‘AND’, ‘OR’ and ‘SCORE’ options allow the user to specify if the program should search for i) SNPs in the motif AND the genomic regions, or ii) SNPs either in the motif OR the genomic regions, or iii) SNPs in regions AND the motif plus in those regions that exceed a user-defined SCORE threshold.

The MAP file describes the genotype data provided by the user. Each line of the file describes a single marker and must contain exactly four columns: chromosome, rs-number, genetic distance (in centimorgan) and bp position.

#### (c) LD files and r^2^-threshold

The user can specify an r^2^-threshold that ReMo-SNPs applies to look for proxy markers in LD-blocks for non-genotyped SNPs. This is valuable for SNPs in interesting genomic regions and/or motifs that are not included in the genotyping platform used for the study.

#### (d) Long, medium and short runs

By default the program is set to run the long version of the analysis, which includes all seven steps of the program. When using ReMo-SNPs to select SNPs for an association study, the user will choose the long run, which generates genome-wide data on markers in the user-specified motifs and/or regions. In addition, a list with interesting markers, for which the program was unable to find genotype data, is also provided. The medium and short runs generate descriptive statistics of the SNPs located in the regions and/or motifs of interest. For both these options the program ends after step 4. The medium run provides descriptive statistics on SNPs located in motifs. The presence of multiple polymorphisms within a short motif may indicate low sequence quality for that part of the genome. The short option provides information on which SNPs are located in motifs and regions of interest, respectively. For all three options the program provides information about how many times a SNP is found in each position of the motif, and how many motifs contain one, two, three or more SNPs.

### (ii) Files to download

Using ReMo-SNPs requires the download of several publicly available data files. Each file, or category of files, should be saved in a separate folder on the local hard drive.

The ReMo-SNPs Perl script can be downloaded here: http://www.neuro.ki.se/ReMo-SNPs/

If the user does not yet have the Perl interpreter installed, it can be downloaded at: http://www.perl.org/get.html

A relevant HapMap file can be downloaded at: http://hapmart.hapmap.org/BioMart/martview. Our example uses genomic Build 36. The resulting text file has three columns: chromosome, position and marker ID.

#### (a) FASTA files

ReMo-SNPs requires one FASTA file for each chromosome. The IUPAC-masked files, which provide information regarding the position of SNPs, can be downloaded from the genome browser at: http://genome.ucsc.edu/. In our example we used SNP129-FASTA, hg18 build 36·1, March 2006. It is absolutely crucial that the FASTA files and the HapMap file are based on the same build.

#### (b) LD files

LD files, containing pairwise LD data, can be downloaded from http://hapmap.org/ then go to Bulk Data Download then go to LD Data. These files are compressed (.gz) and should not be unpacked for the ReMo-SNPs analysis. Since the LD of SNPs varies greatly between populations, it is utterly important that one download LD data for the same population as the one in the user provided genotype data set.

### (iii) Program overview

**Fig. 1.**
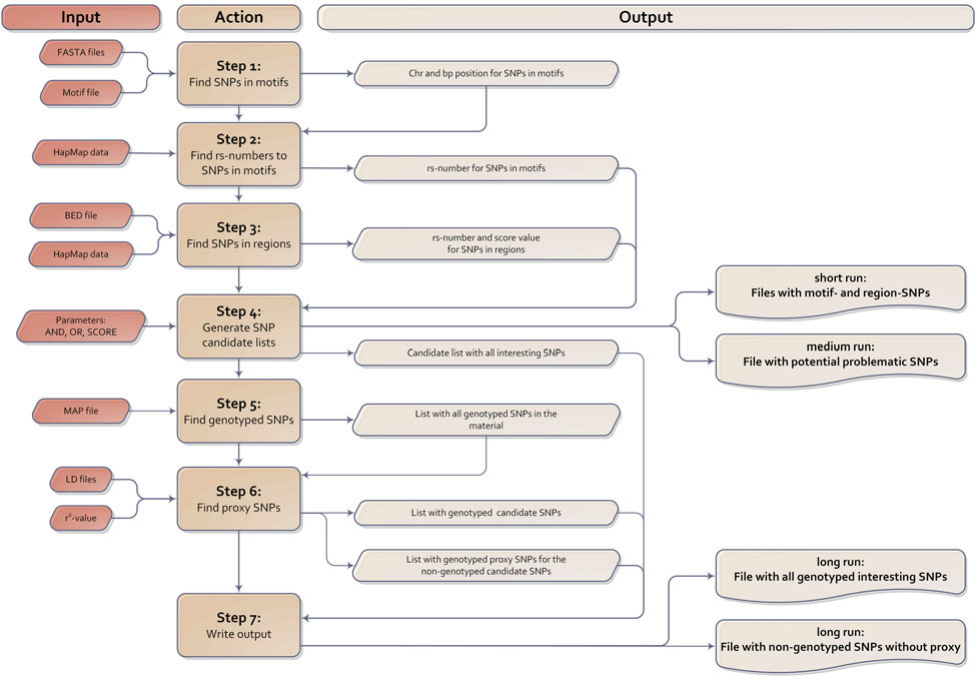
Flowchart illustrating an overview of the input, action and output parts of the ReMo-SNPs program.

The ReMo-SNPs program comprises seven steps, which are described below. An overview of the input, action and output parts of the program is illustrated in a flowchart in Fig. 1. Detailed descriptions of command line options and the contents of the different output files, the log file and information specified in the terminal window are provided in the Appendix.

#### (a) Step 1: find markers in motifs genome-wide

The nucleotide sequences in the FASTA files contain information on which positions are variable. By scanning through each FASTA file step by step the program identifies all instances of the user-provided motif. It then determines whether or not the motif contains any polymorphisms. In parallel, the program gathers descriptive statistics on how many times the motif was found (with or without SNPs), how many times a SNP occurred in each position and how many SNPs each motif contained. Motifs with more than one SNP are potentially problematic, because they may indicate low-quality sequencing data. The script issues a warning about such motifs and provides information about their physical position.

#### (b) Step 2: find rs-numbers for markers from step 1

Step 1 provides information on the position for each motif-SNP, but not the rs-number. By comparing the physical locations with information provided in the HapMap data file, the program identifies the rs-numbers of all HapMap validated SNPs found in step 1.

#### (c) Step 3: find markers in genomic regions of interest

In this step, the program uses the user-provided BED file with information on the genomic regions of interest to identify SNPs located in these regions. Regions without a user-defined score are assigned a default value of 1.

#### (d) Step 4: combine lists according to user input and generate a list of candidate SNPs

The script uses the data generated in steps 2 and 3 to generate candidate lists of SNPs placed in interesting regions and/or motifs. The user-specified AND, OR or SCORE options are used in this step to determine whether to search for SNPs that occur in regions, motifs or both. If the user has chosen to carry out a medium or short run, the script provides descriptive statistical data gathered so far, and the run ends here. In a medium run, the program identifies the rs-numbers for the potentially problematic SNPs identified in step 1 (motifs that contain more than one SNP) and provides information on these SNPs in the terminal window. If the user has chosen a short run, the program writes the SNPs located in regions and/or motifs of interest to two separate output files.

#### (e) Step 5: go through the MAP file and obtain a list of all genotyped markers

In this step the program works on the genotype data file, the MAP file, provided by the user. It goes through this file and extracts information about the genotyped markers in the material.

#### (f) Step 6: find genotyped markers and proxy markers for those markers that have not been genotyped

To identify which of the candidate SNPs identified in steps 1–4 have been genotyped in the material, the program now compares the candidate SNP list created in step 4 with the information generated in step 5. For markers not genotyped in the material the script now searches for proxy SNPs that are in high LD with the SNP of interest. It accomplishes this task by using information in the LD data files. The r^2^-threshold is specified by the user on the command line when starting the script. The identified proxy SNPs are written to a file called lddata.txt. The script then analyses the list of identified LD-markers to see if any of these have been genotyped in the material and could be included in the study to provide information about the original non-genotyped marker. If a candidate SNP has several proxy LD-SNPs, the one with the highest r^2^-value is chosen. The identified genotyped proxy SNPs are written to a file called genotyped.lddata.txt.

#### (g) Step 7: write output

Two output data files are created in this step. The first, called ReMo.SNPs.out, lists all interesting genotyped markers from the candidate list created in step 4 and the genotyped LD-markers from step 6. The second output file, called list.of.markers.with.no.genotype.and.no.proxy.out, lists SNPs that are interesting because of their genomic location in a putative functional region, but have not been genotyped and have no good proxy marker.

### (iv) Transcription factor binding regions

Information on the genome-wide transcription factor binding regions used in this study was downloaded from publicly available data sources. Chromatin immunoprecipitation (ChIP) followed by next-generation DNA-sequencing was used to identify the 15 847 binding regions for the GR reported by Reddy *et al.* (2009), as well as the 2276 VDR binding regions reported by Ramagopalan *et al.* (2010). Both data sets were obtained from the supplemental information of the respective publications. Schmidt *et al.* (2011) reported two data sets with genome-wide PPAR binding including 37 554 and 27 838 binding sites, respectively. We downloaded these data sets from the NCBI Gene Expression Omnibus (GEO) page, with GEO accession number: GSM678397 and GSM678398. We identified overlapping regions between the two data sets and then removed 5% of the biggest regions, which were possible artifacts, as reported in the original study. The remaining 22 456 PPAR binding regions were used in our study.

### (v) Motifs

Homodimers of ligand-bound GR translocate from the cytosol to the nucleus and bind to specific DNA responsive elements called glucocorticoid response elements. In our study we used the GR half-site, RGnACA, identified by Reddy *et al.* (2009). The activated PPAR forms heterodimers with the retinoid X receptor (RXR) before binding at peroxisome proliferator hormone response elements on the DNA. We used a minimal PPAR-motif, AGGTCA, which has been reported in several studies (IJpenberg *et al.*, 1997; Juge-Aubry *et al.*, 1997; Michalik *et al.*, 2006; Degenhardt *et al.*, 2007). Like PPAR, VDR also forms heterodimers with RXR before binding to hormone response elements on the DNA. Since the full VDR motif, AAGGTCAnAGAGTTCA, reported by Ramagopalan *et al.* (2010), is very long and specific, we instead used the minimal motif, RGKKSA, reported by several groups (Heikkinen *et al.*, 2011; Hidalgo *et al.*, 2011; Zhang *et al.*, 2011; Meyer *et al.*, 2012).

### (vi) SNP density analysis

To obtain information about the distribution of the SNPs identified by the ReMo-SNPs program, we calculated the densities of SNPs in the regions and motifs of interest.

#### (a) Region- and motif-SNP density

For each transcription factor the SNP density was calculated by dividing the total number of SNPs in regions of interest by the total length of all regions for that transcription factor. For each transcription factor the motif-SNP density was calculated by dividing the total number of SNPs found in the motif of interest by the total length of the motifs, which was calculated by multiplying the motif length in bases by the total number of motifs found in the genome. To obtain a comparison number for the entire human genome, we divided the total number of SNPs reported in the HapMap file for Utah residents with Northern and Western European ancestry (CEU) from the CEPH collection (2 814 954 SNPs) by the total number of bps in the entire genome (given by the total length of the FASTA files).

### (vii) SNP distribution within the motifs

The SNP distribution within the motifs was analysed in two ways: i) total number of SNPs in each position of the motif and ii) the distribution of motifs with 1, 2, 3 or more SNPs. These descriptive data were generated by running the short version of the ReMo-SNPs software on each one of the three transcription factors.

### (viii) External assessment of functional SNPs

In order to evaluate the power of the ReMo-SNPs software to identify functional SNPs we compared the output data generated from motif-SNPs placed within the transcription factor regions of interest (SNPs in a motif placed within any of the regions defined in the BED file) with those outside these regions (SNPs in a motif not placed within any of the regions defined in the BED file). No gold standard currently exists, and each computational tool has unique strengths and weaknesses. The identified SNPs for each data set were, therefore, tested using three different software tools that calculate functionality scores for the SNPs: Regulome (http://regulome.stanford.edu/), SNP Function Annotation Portal (http://brainarray.mbni.med.umich.edu/Brainarray/Database/SearchSNP/snpfunc.aspx) and SNP Function Prediction (http://snpinfo.niehs.nih.gov/snpinfo/snpfunc.htm). For each program the generated functional scores were translated to numerical values and added together for 75 randomly chosen SNPs from each data set to generate an average value.

### (ix) Quality control of the association study materials

The quality control analyses as well as the following association analyses and statistical calculations were performed with the open-source software PLINK (http://pngu.mgh.harvard.edu/~purcell/plink/) as previously described (Graae *et al.*, 2012).

### (x) Association analysis

A two-tailed Fisher's exact test was performed for the association studies. Statistical significance was defined as p < 0·05 applying Bonferroni correction for multiple testing.

## 4. Results

### (i) Program output data

The long version of ReMo-SNPs was run for all three transcription factors for both the MD and the BP material. The generated output data are shown in Table 2.

**Table 2.** Results from the long run with the ReMo-SNPs program.

	GR		PPAR		VDR	
	MD	BP	MD	BP	MD	BP
Region-SNPs	8207	8207	11 712	11 712	2656	2656
Motif-SNPs	136 925	136 925	16 287	16 287	216 412	216 412
Candidate SNPs	545	545	85	85	255	255
Genotyped candidate SNPs	91	129	14	18	48	58
LD-SNPs	3667	3322	653	545	1656	1439
Genotyped LD-SNPs	228	201	32	36	91	84
SNPs without genotype or LD-marker	226	215	39	31	116	113
Total number of genotyped interesting SNPs	319	330	46	54	139	142
SNPs excluded during quality control	4	4	0	0	6	3
SNPs in association study	315	326	46	54	133	139

Approximately 8200 SNPs were found in the GR regions, 11 700 SNPs in the PPAR regions and 2650 SNPs in the VDR regions. The program found approximately 137 000 SNPs in the GR motif genome-wide, 16 300 SNPs in PPAR motifs and 216 400 SNPs in VDR motifs. When combining this data and searching for motif-SNPs placed within the experimentally validated binding regions the program found 545 such SNPs in the GR data set, 85 in the PPAR data set and 255 in the VDR data set. The ReMo-SNPs program then identified which of these SNPs were genotyped in the user provided material and tried to find SNPs in high LD for the not genotyped SNPs. In total there were approximately 320 genotyped GR SNPs, approximately 50 genotyped PPAR SNPs and approximately 140 genotyped VDR SNPs. A few of these SNPs were excluded during the quality control steps and in the end there were 315 GR SNPs, 46 PPAR SNPs and 133 VDR SNPs in the MD material, and 326 GR SNPs, 54 PPAR SNPs and 139 VDR SNPs in the BP material that could be included in the association analysis.

The runtime for the program varies from seconds to several hours depending on several aspects such as: type of run (e.g. short, medium or long); if the user chooses to analyse the data genome-wide or only in one chromosome; the number of regions and type of motif. When running the long version of the program with genome-wide data as described above the analyses took a few hours to complete.

### (ii) Quality control of the material

A summary of individuals and SNPs excluded in the different quality control steps as described in the Methods section are shown in Table 3.

**Table 3.** Summary of individuals and SNPs excluded in each quality control step.

Data set	Test	Threshold	Number of excluded individuals or SNPs	Number of individuals or SNPs before test	Number of individuals or SNPs after test
MD	Missing rate per person	>0·1	0	3485	3485
	Hardy-Weinberg equilibrium	⩽0·0001	720	438 129	437 409
	Minor allele frequency	<0·01	68	437 409	437 341
	Missing genotypes	>0·05	408	437 341	436 933
BP	Missing rate per person	>0·1	0	1962	1962
	Hardy-Weinberg equilibrium	⩽0·0001	39	650 635	650 596
	Minor allele frequency	<0·01	0	650 596	650 596
	Missing genotypes	>0·05	73	650 596	650 523

### (iii) Association analysis

None of the genotyped candidate SNPs remained significant after correcting for multiple testing. Table 4 shows the top associated SNP for each data set.

**Table 4.** Association results showing the top associated SNP for each data set.

TF	Disease	Gender	Number of SNPs	rs-number	Alleles^*a*^	MAF (cases)	MAF (controls)	p-value^*b*^	corrected p-value^*c*^
GR	MD	All	315	rs7802018	G < A	0·3294	0·3675	0·0008899	>1
		Females	315	rs7802018	G < A	0·3241	0·0373	0·0006633	>1
		Males	315	rs4820741	T < C	0·1705	0·2186	0·0032550	>1
	BP	All	326	rs1891805	G < A	0·0494	0·0732	0·0021650	>1
		Females	326	rs6696816	C < T	0·4220	0·3439	0·0004041	>1
		Males	326	rs2284933	C < G	0·3742	0·4518	0·0005050	>1
PPAR	MD	All	46	rs6052286	G < T	0·2967	0·2688	0·0105700	>1
		Females	46	rs6052286	G < T	0·3038	0,2698	0·0115100	>1
		Males	46	rs1918778	C < T	0·2380	0·2862	0·0089750	>1
	BP	All	54	rs13142632	G < C	0·3250	0·3601	0·0220000	>1
		Females	54	rs13142632	G < C	0·3066	0·3671	0·0054350	>1
		Males	54	rs6844643	G < A	0·3522	0·2963	0·0080810	>1
VDR	MD	All	133	rs178399	A < G	0·4394	0·4086	0·0099420	>1
		Females	133	rs178399	A < G	0·4515	0·4110	0·0062530	>1
		Males	133	rs3011770	C < T	0·2643	0·3118	0·0113700	>1
	BP	All	139	rs3100610	C < T	0·2749	0·2425	0·0215700	>1
		Females	139	rs9516887	C < T	0·4127	0·4714	0·0094340	>1
		Males	139	rs4142872	A < C	0·2564	0·3120	0·0068560	>1

a Minor allele < major allele.

b The lowest p-value for each group of association tests.

c p-value corrected for 3039 markers.

MAF, minor allele frequency; TF, transcription factor.

### (iv) External assessment of functional SNPs

We evaluated the power of ReMo-SNPs to identify functional SNPs by using three different functional software tools: Regulome, SNP Function Annotation Portal and SNP Function Prediction (Wang *et al.*, 2006; Xu & Taylor, 2009; Boyle *et al.*, 2012). By analysing the SNPs identified by the ReMo-SNPs program with these tools we obtained external scores for how likely it was that a certain ReMo-identified SNP would be functional. This validation method included ReMo-identified motif-SNPs located within vs. outside experimentally verified binding regions.

#### (a) Motif-SNPs placed within vs. outside transcription factor binding regions

**Fig. 2.**
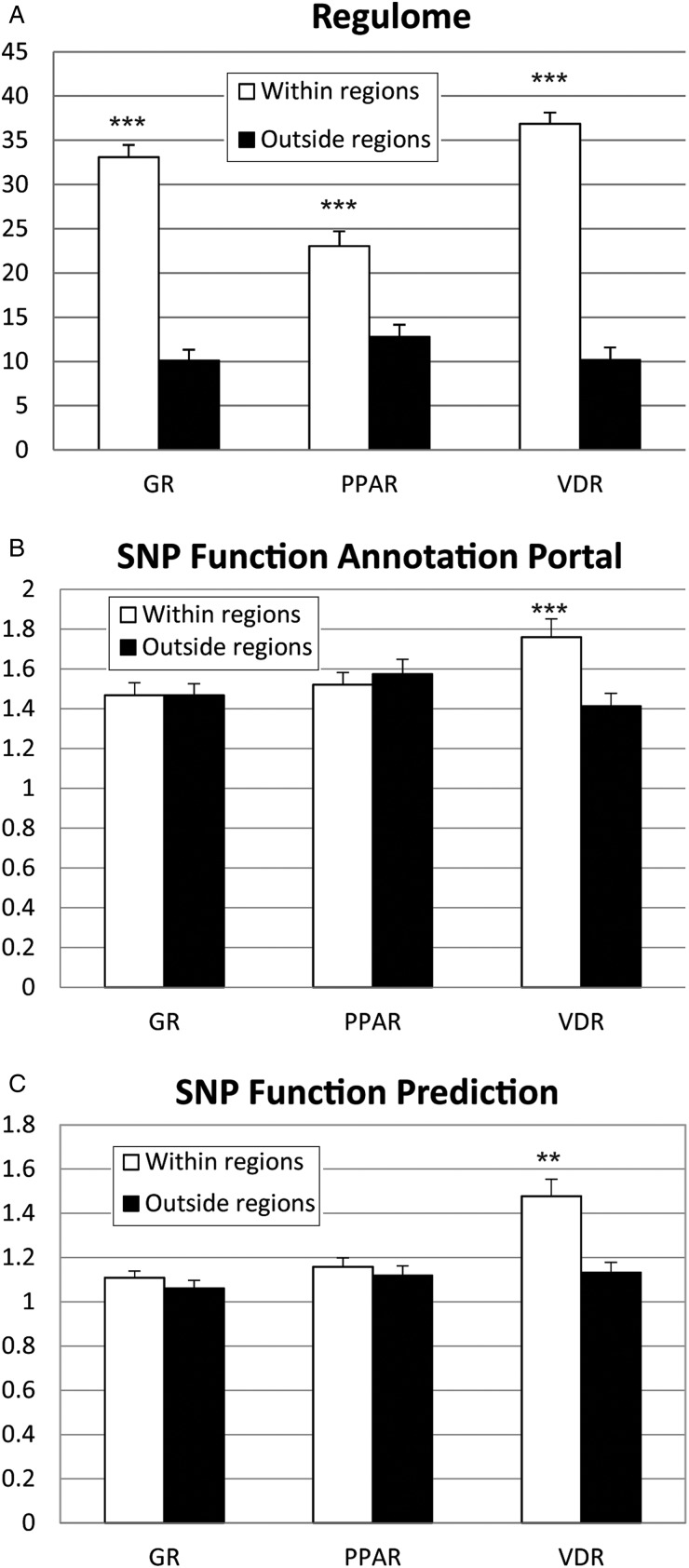
Motif-SNPs placed within vs. outside experimentally verified transcription factor binding regions for (*a*) Regulome, (*b*) SNP Function Annotation Portal and (*c*) SNP Function Prediction. The score-values on the y-axis are unique for each program and could therefore not be compared between the different programs. Data is presented as mean ± standard error of the mean, ** = p < 0·01, *** = p < 0·0001.

The VDR motif generated higher functional average scores for motif-SNPs placed within experimentally verified binding regions compared to outside these regions in all assessment tools (p < 0·01, using Student's t-test, see Fig. 2). The GR and PPAR motifs generated higher functional average scores in one out of the three validation programs (p < 0·000·1, Regulome). Note: the score-values on the y-axis are unique for each functional program and should thus not be compared between the different tools.

### (v) SNP density analysis

**Fig. 3.**
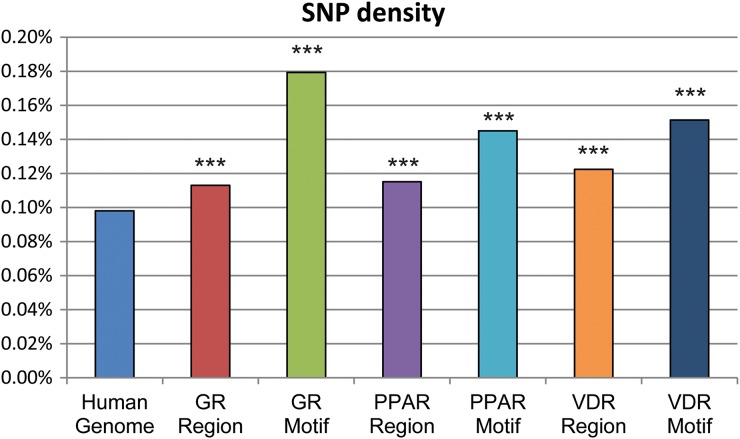
Assessment of SNP densities in regions and motifs of interest compared to the genome at large. Average SNP density in the human genome of the CEU population and in the binding regions and motifs for the three transcription factors, GR, PPAR and VDR. Data is presented as *** = p < 0·0001.

The density of SNPs in the transcription factor binding regions was calculated and compared to the average SNP density in the human genome, based on data from CEU ancestry from the CEPH collection. All three transcription factor binding regions showed a higher SNP density compared to the average SNP density in the CEU human genome (p < 0·0001, using Chi-square test). VDR and PPAR had a SNP density close to 0·12%, GR just above 0·11% and the CEU human genome just below 0·10% (see Fig. 3). In addition to studying the SNP density in the transcription factor binding regions, we also calculated the SNP density in the transcription factor motifs. Compared to the average SNP density in the human genome, the SNP density for the motifs was significantly higher (p < 0·0001). The GR motif had the highest SNP density with 0·18% whereas PPAR and VDR both had 0·15%.

### (vi) SNP distribution within the motifs

**Fig. 4.**
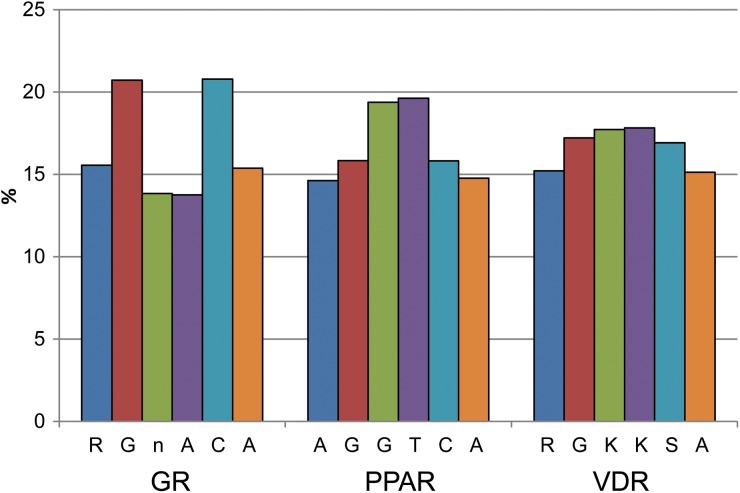
The distribution of SNPs found at different positions within the motif. The bars represent the six different nucleotide positions within the motifs and the y-axis shows the amount of SNPs in percent found for each position normalized to the total number of SNPs found for each motif. n = any nucleotide, A, T, G or C; R = A or G; K = T or G; and S = C or G.

The total number of SNPs at different positions within the motifs and for each type of nucleotide was analysed. The numbers were normalized to the total amount of SNPs found for each motif. No consistent pattern was observed for any of the three studied transcription factors (see Fig. 4). It is well known that different types of nucleotides mutate at different rates, with G and C having a higher mutation rate than A and T. It was, therefore, surprising to see the high mutation rate for T (19·6%) in the PPAR motif vs. G (15·8 and 19·4%) and C (15·8%). As expected, the G and C nucleotides together (37·3%) generally harboured more SNPs compared to A and T together (21·1%).

We further analysed the SNP distribution for each type of nucleotide.

**Fig. 5.**
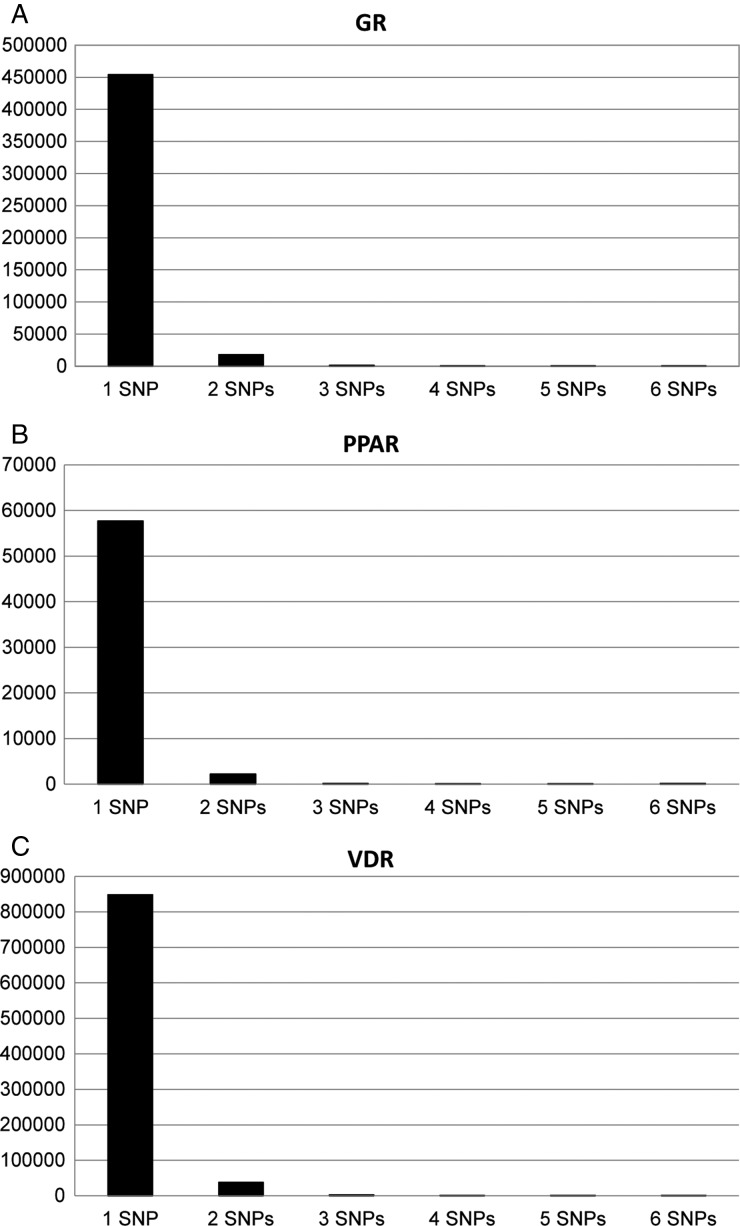
The number of SNPs found per motif for each transcription factor (*a*) GR, (*b*) PPAR and (*c*) VDR.

A final descriptive analysis of the number of SNPs per motif showed that very few of the motifs were polymorphic and that the vast majority of all of the variable transcription factor motifs only had one SNP per motif (see Fig. 5(*a–c*)).

## 5. Discussion

Recent advances in genomic technologies have enabled researchers to gather enormous amounts of data for the study of genetically complex traits. A substantial part of the genetic contribution to these traits is likely being accounted for by non-coding, regulatory variants. This insight has driven a strong need for ever-more sophisticated bioinformatics tools to deal with the resulting large data sets. Furthermore, in addition to performing GWAS with all genotyped markers, more targeted approaches that investigate pathways and regulatory networks are emerging. To meet the demand for tools dedicated to the genome-wide analysis of non-coding, regulatory variation, we have developed ReMo-SNPs. We herein describe its functionality and compare its output to other available computational programs.

### (i) Identification and external assessment of functional SNPs

We evaluated ReMo-identified motif-SNPs within and outside experimentally verified transcription factor binding regions using three different software tools (Regulome, SNP Function Annotation Portal and SNP Function Prediction). This step generated functionality scores for each SNP found by ReMo-SNPs based on previous reported data in different databases.

SNPs located within experimentally verified transcription factor binding regions generated significantly higher scores for all three transcription factors in the Regulome assessment tools. For the SNP Function Annotation Portal and SNP Function Prediction tools, VDR motif-SNPs within verified regions generated significantly higher scores, the rest showed a tendency for higher scores except for PPAR in the SNP Function Annotation Portal, where the motif-SNPs placed outside the transcription factor binding regions generated higher scores. These results illustrate the benefit of being able to combine *in silico* identified motif-SNPs with experimentally validated transcription factor binding regions, which leads to an enrichment for functional variants in the target data set.

### (ii) SNP density analyses

Compared to the average SNP density in the human genome for the CEU population (just below 0·10%), the density of SNPs was higher in the transcription factor binding regions (0·11–0·12%) and even higher within the motif sequences (0·15–0·18%), which is in very good agreement with results from previous studies (Guo & Jamison, 2005; Vernot *et al.*, 2012). Guo and Jamison, for example, found an overall SNP density of 0·13% in gene promoter regions, which increased to 0·20% in predicted transcription factor binding site regions.

The uneven distribution of SNPs within the genome has long been known to mirror the evolutionary pressure on different regions, with fewer SNPs found in exons compared to introns and pseudogenes, where the evolutionary pressure is lower. Since it is more likely that a SNP causes a deleterious effect when placed inside an exon compared to an intron, natural selection keeps sorting out SNPs in exons. Why then do we find a higher SNP density in the regulatory regions compared to the average sequences in the genome? One explanation could be that SNPs placed in regulatory regions may enable a more fine-tuned response to environmental challenges. The ability to adjust gene regulation by slightly altering gene-expression levels might be of major importance for species to adapt to ever-changing environments throughout evolution.

### (iii) Motif-SNP distribution analyses

The motif-SNP distribution was analysed in several ways: number of SNPs at different positions within the motifs and for each nucleotide in the motif; as well as number of SNPs per motif. When analysing the number of SNPs at different positions in the motifs, no consistent pattern could be observed. One would expect that a SNP located in the flanking regions of the motif would have a smaller effect on the transcription factor binding properties compared to a SNP placed in the middle of the motif sequence. In that case we would have seen more SNPs in the flanking regions and fewer SNPs in the middle parts. For the motifs of both PPAR and VDR the opposite pattern was observed instead, which suggests that the process of SNP distribution is far more complex, perhaps reflecting the different mutation rates for different nucleotides in combination with the probable different mutation tendencies at different positions within the motif.

The SNP distribution for each type of nucleotide showed that, as expected, the G and C nucleotides harboured more SNPs compared to A and T. Known regulatory regions have increased CpG rates (also known as CpG islands). This observation is, therefore, in agreement with the above-mentioned finding that the functional regions harbour more SNPs than the genome at large.

We also analysed the number of SNPs per motif. Considering that the motif sequence length was only six nucleotides long it is not surprising that very few motifs had more than one SNP.

### (iv) Association results

The association studies did not generate significant p-values after correcting for multiple testing. We chose the GR, PPAR and VDR transcription factors in this study mainly because of practical reasons regarding the availability of high-quality genome-wide experimentally validated binding data. There was no particular *a priori* correlation of these markers with MD or BP. It will be interesting to follow up on this work by looking at transcription factors and gene regulatory networks that have emerged recently for these diseases. ReMo-SNPs can be a valuable tool to help researchers with these studies.

## 6. Conclusions

We herein introduce a new computational tool that can be used to enrich genetic data sets for predicted functional variants. ReMo-SNPs can quickly analyse genome-wide data and combine input from *in silico* and *in vitro* analyses. We believe that the flexibility and user-friendliness of ReMo-SNPs will be very helpful to researchers who want to select functional SNPs for association analyses in user-specified regions and/or motifs genome-wide.

## Appendix

1. Detailed descriptions on command line options, output files and terminal window output

### (i) Command line

On the command line the user specifies the required information for each type of run. For a long run all information, a) – n), should be provided. For medium and short runs, the user should specify the information stated in a) – h) below.

a) perl -w ReMo.SNPs.pl
b) – HapMap [path and name of the HapMap data file]
c) – Motifs [path and name of the motif file]
d) – FASTAdir [path and name of the folder with FASTA files]
e) – bed [path and name of the BED file containing the region data]
f) – regionScore [value for the score-threshold; this command is optional]
g) – combo [AND, OR or SCORE, for type of combination of regions and motifs]
h) – typeOfRun [long, medium or short. The default value is ‘long’ for a full run]
i) – map [path and name of the MAP file]
j) – LDdir [path and name of the folder with the .gz LD files; do not unzip these files for analysis]
k) – r^2^ [between 0·0 and 1·0; threshold for inclusion of proxy markers]
l) – log [file name for the log-file (the default name is ReMo.SNPs.log)]
m) – out [file name for the out-file (the default name is ReMo.SNPs.out)]
n) >name.of.screenoutput.file.txt, optional command to re-direct the script's output if the user wants to save the information written in the terminal window

### (ii) Output files and information

#### (a) ReMo.SNPs.out

This file is created in step 7. It shows all interesting genotyped markers from the candidate list in step 4 and the genotyped LD-markers from step 6.

#### (b) lddata.txt-file

This file is created in step 6 when the script searches for proxy markers for those markers that have not been genotyped. It contains 11 columns with the following information: chromosomal position of marker 1, chromosomal position of marker 2, population code, rs-number for marker 1, rs-number for marker 2, D′, R^2^, LOD, fbin, rs-number of the candidate SNP and chromsome.

#### (c) Genotyped.lddata.txt-file

This file is also created in step 6, when the program identifies SNPs from the lddata.txt-file that have been genotyped in the material. It contains the same columns as the lddata.txt-file.

#### (d) List.of.markers.with.no.genotype.and.no.proxy.out

This file shows the interesting SNPs from the candidate list that should be analysed based on their location but have not been genotyped and have no good LD-SNP.

#### (e) motifsnplist.txt and regionsnplist.txt

These files are created in step 4 if one has chosen the short run. They show all the SNPs found to be located in the motif of interest genome-wide and the specified regions of interest, respectively.

### (iii) Log file example output

This is ReMo.SNPs.pl

Analysis started with the following arguments:

(In step 1:)

Currently working on Chromosome A

Sequence length and line counter

The sequence length shows how many letters the FASTA file contains and the line counter corresponds to the number of rows the FASTA file had before the program made one row of it.

After giving this information for all chromosomes, the script provides information for each chromosome on how many SNPs are found to be located in the motif of interest.

(In step 2:)

Information on how many markers the program found genome-wide in the motif of interest is given.

(In step 3:)

Information on how many markers the program found in genomic regions of interest is given.

(In step 4:)

The number of total candidate SNPs is given.

(In step 5:)

The number of markers that have been read from the MAP file is printed.

(In step 6:)

The number of interesting SNPs with genotypes is given.

### (iv) Terminal window example output

Step 1 …

My motif is X character long

The original motif is ABC…

The reverse complement is ABC…

The IUPAC-motif is: [ABC…][ABC…]…

The reverse IUPAC-complement is: [ABC…][ABC…]…

Problem SNP found: chr/bp/motif-length

Position 1 had A mutations

Position 2 had B mutations

…

There were C motifs with one SNP(s)

There were D motifs with two SNP(s)

…

Step 2…

No rs-number was found for the following sequence: XXX at position YYY on chromosome ZZ

Step 3…

Step 4…

If a medium run is chosen the following will be printed in this step:

HapMap: position in bp and rs-number problem: position in bp and motif-length

The following SNPs may be problematic because they are located in motifs with more than one SNP:

Step 5…

Step 6…

Step 7…

## Notes

This study was supported by the Swedish Research Council (A.C.B., K2013-99X-22248-01-3); the Swedish Brain Foundation (A.C.B., grant number FO2013-0213); Swedish Brain Power (A.C.B., grant number SBP II); Åke Wibergs Stiftelse (A.C.B., grant number 352107435); the Swedish Parkinson Foundation (A.C.B., grant number 613/13) and Karolinska Institutet research funds (A.C.B., grant number 2014fobi41360). The authors would also like to thank Robert Karlsson PhD for valuable input.

Funding support for the Genetic Association Information Network (GAIN) Major Depression: Stage 1 Genome-wide Association In Population Based Samples Study (parent studies: the Netherlands Study of Depression and Anxiety (NESDA) and the Netherlands Twin Register (NTR)) was provided by the Netherlands Scientific Organization (904-61-090,904-61-193,480-04-004,400-05-717, NWO Genomics, SPI 56–464–1419) the Centre for Neurogenomics and Cognitive Research (CNCR-VU); the European Union (EU/WLRT-2001-01254), ZonMW (geestkracht program, 10-000-1002), NIMH (RO1 MH059160) and matching funds from participating institutes in NESDA and NTR, and the genotyping of samples were provided through GAIN. The data set(s) used for the analyses described in this manuscript were obtained from the database of Genotypes and Phenotypes (dbGaP) found at http://www.ncbi.nlm.nih.gov/gap through dbGaP accession number phs000020.v2.p1. Samples and associated phenotype data for the GAIN Major Depression: GAIN March 1, 2010 version Page 9 of 11 Stage 1 Genome-wide Association In Population Based Samples Study (PI: Dr Patrick F. Sullivan, MD, University of North Carolina) were provided by Dr Dorret I. Boomsma, PhD and Dr Eco de Geus, PhD VU University Amsterdam (PIs NTR), Dr Brenda W. Penninx, PhD, VU University Medical Center Amsterdam, Dr Frans Zitman, MD, PhD, Leiden University Medical Center, Leiden and Dr Willem Nolen, MD, PhD, University Medical Center Groningen (PIs and site PIs NESDA).

Whole Genome Wide Association Study of Bipolar Disorder Funding support for the Whole Genome Association Study of Bipolar Disorder was provided by the National Institute of Mental Health (NIMH) and the genotyping of samples was provided through GAIN. The data sets used for the analyses described in this manuscript were obtained from the database of Genotypes and Phenotypes (dbGaP) found at http://www.ncbi.nlm.nih.gov/gap through dbGaP accession number phs000017.v3.p1. Samples and associated phenotype data for the Collaborative Genomic Study of Bipolar Disorder were provided by the NIMH Genetics Initiative for Bipolar Disorder. Data and biomaterials were collected in four projects that participated in NIMH Bipolar Disorder Genetics Initiative. From 1991–98, the Principal Investigators and Co-Investigators were: Indiana University, Indianapolis, IN, U01 MH46282, John Nurnberger, MD, PhD, Marvin Miller, MD and Elizabeth Bowman, MD; Washington University, St. Louis, MO, U01 MH46280, Theodore Reich, MD, Allison Goate, PhD and John Rice, PhD; Johns Hopkins University, Baltimore, MD U01 MH46274, J. Raymond DePaulo, Jr, MD, Sylvia Simpson, MD, MPH and Colin Stine, PhD; NIMH Intramural Research Program, Clinical Neurogenetics Branch, Bethesda, MD, Elliot Gershon, MD, Diane Kazuba, BA and Elizabeth Maxwell, MSW. Data and biomaterials were collected as part of ten projects that participated in the NIMH Bipolar Disorder Genetics Initiative. From 1999–03, the Principal Investigators and Co-Investigators were: Indiana University, Indianapolis, IN, R01 MH59545, John Nurnberger, MD, PhD, Marvin J. Miller, MD, Elizabeth S. Bowman, MD, N Leela Rau, MD, P. Ryan Moe, MD, Nalini Samavedy, MD, Rif El-Mallakh, MD (at University of Louisville), Husseini Manji, MD (at Wayne State University), Debra A. Glitz, MD (at Wayne State University), Eric T. Meyer, MS, Carrie Smiley, RN, Tatiana Foroud, PhD, Leah Flury, MS, Danielle M. Dick, PhD and Howard Edenberg, PhD; Washington University, St. Louis, MO, R01 MH059534, John Rice, Ph.D, Theodore Reich, MD, Allison Goate, PhD and Laura Bierut, MD; Johns Hopkins University, Baltimore, MD, R01 MH59533, Melvin McInnis MD, J. Raymond DePaulo, Jr, MD, Dean F. MacKinnon, MD, Francis M. Mondimore, MD, James B. Potash, MD, Peter P. Zandi, Ph.D, Dimitrios Avramopoulos, and Jennifer Payne; University of Pennsylvania, PA, R01 MH59553, Wade Berrettini MD, PhD; University of California at Irvine, CA, R01 MH60068, William Byerley MD and Mark Vawter MD; University of Iowa, IA, R01 MH059548, William Coryell MD and Raymond Crowe MD; University of Chicago, IL, R01 MH59535, Elliot Gershon, MD, Judith Badner PhD, Francis McMahon MD, Chunyu Liu PhD, Alan Sanders MD, Maria Caserta, Steven Dinwiddie MD, Tu Nguyen and Donna Harakal; University of California at San Diego, CA, R01 MH59567, John Kelsoe, MD and Rebecca McKinney, BA; Rush University, IL, R01 MH059556, William Scheftner MD, Howard M. Kravitz, DO, MPH, Diana Marta, BS, Annette Vaughn-Brown, MSN, RN and Laurie Bederow, MA; GAIN March 1, 2010versionPage 10of 11NIMH Intramural Research Program, Bethesda, MD, 1Z01MH002810-01, Francis J. McMahon, MD, Layla Kassem, PsyD, Sevilla Detera-Wadleigh, PhD, Lisa Austin, PhD and Dennis L. Murphy, MD.
